# Workplace violence in a large correctional health service in New South Wales, Australia: a retrospective review of incident management records

**DOI:** 10.1186/1472-6963-12-245

**Published:** 2012-08-09

**Authors:** Aaron W Cashmore, Devon Indig, Stephen E Hampton, Desley G Hegney, Bin B Jalaludin

**Affiliations:** 1 New South Wales Public Health Officer Training Program, New South Wales Ministry of Health, North Sydney, New South Wales, Australia; 2 Justice Health, Matraville, New South Wales, Australia; 3 School of Public Health and Community Medicine, University of New South Wales, Kensington, New South Wales, Australia; 4 School of Medicine, University of Western Sydney, Campbelltown, New South Wales, Australia; 5 Curtin Health Innovation Research Institute, Curtin University, Perth, Western Australia, Australia; 6 Centre for Research, Evidence Management and Surveillance, South Western Sydney Local Health District, Liverpool, New South Wales, Australia; 7 Centre for Nursing Research, Sir Charles Gairdner Hospital, Perth, Western Australia, Australia

**Keywords:** Workplace violence, Correctional health professionals, Incident management

## Abstract

**Background:**

Little is known about workplace violence among correctional health professionals. This study aimed to describe the patterns, severity and outcomes of incidents of workplace violence among employees of a large correctional health service, and to explore the help-seeking behaviours of staff following an incident.

**Methods:**

The study setting was Justice Health, a statutory health corporation established to provide health care to people who come into contact with the criminal justice system in New South Wales, Australia. We reviewed incident management records describing workplace violence among Justice Health staff. The three-year study period was 1/7/2007-30/6/2010.

**Results:**

During the period under review, 208 incidents of workplace violence were recorded. Verbal abuse (71%) was more common than physical abuse (29%). The most (44%) incidents of workplace violence (including both verbal and physical abuse) occurred in adult male prisons, although the most (50%) incidents of physical abuse occurred in a forensic hospital. Most (90%) of the victims were nurses and two-thirds were females. Younger employees and males were most likely to be a victim of physical abuse. Preparing or dispensing medication and attempting to calm and/or restrain an aggressive patient were identified as ‘high risk’ work duties for verbal abuse and physical abuse, respectively. Most (93%) of the incidents of workplace violence were initiated by a prisoner/patient. Almost all of the incidents received either a medium (46%) or low (52%) Severity Assessment Code. Few victims of workplace violence incurred a serious physical injury – there were no workplace deaths during the study period. However, mental stress was common, especially among the victims of verbal abuse (85%). Few (6%) victims of verbal abuse sought help from a health professional.

**Conclusions:**

Among employees of a large correctional health service, verbal abuse in the workplace was substantially more common than physical abuse. The most incidents of workplace violence occurred in adult male prisons. Review of the types of adverse health outcomes experienced by the victims of workplace violence and the assessments of severity assigned to violent incidents suggests that, compared with health care settings in the community, correctional settings are fairly safe places in which to practice.

## Background

Violence is a major global public health problem. According to the World report on violence and health [1], in 2000, the global estimate of violence-related deaths was 1.6 million. Almost certainly an underestimate, this figure represents the tip of the iceberg when considered in relation to non-fatal violent incidents [1]. Although we tend to think of violence as something that occurs in the home or community, violence in the workplace is common [2,3] and incidents often go unreported [4,5].

The health industry includes a variety of institutions (such as hospitals, community health centres and nursing homes) and providers (such as doctors, allied health professionals and nurses). It is one of the most violent industries in which to work [3,6,7]. Numerous studies have found high levels of workplace violence among health workers [8-12], prompting professional bodies such as the International Council of Nurses to actively condemn and advocate the prevention of this aspect of working life [13]. Further, recent research suggests that, despite the proliferation of policies of “Zero Tolerance of Violence”, the occurrence of violence in the health sector has remained steady over the last 10 ;years [14].

The Joint Programme on Workplace Violence in the Health Sector defines workplace violence as: *“Incidents where staff are abused, threatened or assaulted in circumstances related to their work, including commuting to and from work, involving an explicit or implicit challenge to their safety, well-being or health”*[15]. Such incidents may take the form of physical or non-physical abuse and may be initiated by professional colleagues, non-professional staff, employers, patients/clients/residents or visitors.

The consequences of workplace violence for victims are well documented and include a range of physical health and psychosocial problems. Examples of mental health problems that can develop in the victims of workplace violence include anxiety, stress, a feeling of helplessness and, in rare cases, suicidal ideation [14,16-20]. Although its impact on patient care has not been directly measured, a number of self-report studies have found that violence in health care settings likely undermines the quality of health services provision [11,16,17,20-23] – by contributing to clinical errors, for example [11,23]. Clinical errors can happen when a victim of workplace violence (a health professional) worries excessively and/or experiences difficulty concentrating as a result of the incident(s) [11,23]. Further, the perception of an unsafe workplace has been found to lower staff morale and lead to increased staff attrition [11,14,21,24]. Additional implications of workplace violence for employers of health professionals include staff absenteeism, reduced productivity and workers compensation costs [16,17,19-21,23,25].

Levels, patterns and sources of workplace violence have been investigated in most healthcare settings and among most health professions [11,14,16,23,25-31]. However, little is known about workplace violence as it is experienced by nurses and other health professionals who deliver care to prisoners and other people who come into contact with the criminal justice system, although a few studies have explored workplace violence among mental health professionals who were practising in forensic psychiatric facilities [32,33]. A recent cross-sectional survey undertaken by the same research team found a three-month period prevalence of physical abuse among correctional health professionals of 16% [23], which, although unacceptably high, was lower than comparable studies set in the community (proportions of 50% [22] and 30% [11] were found in two Australian studies). Verbal abuse among correctional health workers, on the other hand, was found to be relatively high (76%) [23] – a comparable study of Australian nurses who were working in a variety of settings found a prevalence of verbal abuse of 63% [11].

In order to augment and supplement the self-reported prevalence data produced by the afore-mentioned survey [23], we reviewed three years of routinely collected administrative data on workplace violence among employees – health professionals and non-health staff – of a large correctional health service in New South Wales (NSW), Australia. The aims were to describe the patterns, severity and outcomes of violent incidents, and to explore the help-seeking behaviours of staff following an incident. Such information will be helpful in informing future efforts to prevent and effectively manage workplace violence in correctional settings.

## Methods

### Setting

The study setting was Justice Health, a NSW Government funded statutory health corporation established to provide health care to people, including both adults and juveniles, who come into contact with the criminal justice system in NSW. In 2011, the average daily number of prisoners and detainees in full-time custody in the NSW correctional system was 9,945 [34].

Presently, Justice Health employs over 1,490 people [35]. These employees work in a range of settings, including: police holding cells; adult prisons; periodic detention and transitional centres; a prison hospital; a forensic hospital (opened February 2009); juvenile justice centres; a youth drug and alcohol court; adult and children’s courts; and in the community. The types of health services provided are equally diverse and include: clinical and nursing care; mental health and drug and alcohol services; oral health services; and a range of primary health care services. Eighty per cent of Justice Health staff are health professionals [35]. In line with its strong emphasis on primary care, disease prevention and health promotion, nurses comprise 64% of all staff [35]. Medical doctors make up 5% and allied health professionals make up 3% of total staff [35]. Justice Health operates independently of Corrective Services NSW, which *“provides custodial and community-based services as an important element of the criminal justice system”*[36]. This is an important point to highlight as correctional officers are at an extremely high risk of workplace violence [3,6,32].

A high proportion of Justice Health clients have poor health and complex healthcare needs [37]. Further, many of the individual and socio-political determinants of violence are disproportionately common in prisoner populations [37,38]. The organisation therefore prioritises the work safety of its employees, which is evidenced by the existence of a range a workplace policies, procedures and systems to reduce the risk of workplace violence among staff, including a policy of zero tolerance of violence in the workplace [39], a framework for effective incident management and prevention [40] and the Incident Information Management System (IIMS) [41].

IIMS is an electronic information management system. It is currently used throughout the public health system in NSW, including by Justice Health, to aid in the identification, management and future prevention of health care “incidents”, an incident being *“any unplanned event resulting in, or with the potential to result in, death, injury, ill health, damage or other loss”*[40]. This includes incidents of patient, correctional officer or visitor-initiated workplace violence perpetrated against health professionals or non-health staff. The NSW Ministry of Health and Justice Health policy “Zero Tolerance Response to Violence in the NSW Health Workplace” [39] defines workplace violence as: *“…any incident in which employees are abused, threatened or assaulted in circumstances arising out of, or in the course of their employment. Incidents include verbal, physical or psychological abuse, threats or other intimidating behaviours, intentional physical attacks, aggravated assault, threats with an offensive weapon, sexual harassment and sexual assault.”* Persons who assault employees of Justice Health can be charged with offences under the NSW *Crimes Act 1900*. In order to ensure that the more severe incidents are investigated and managed in a timely way, each IIMS record is assigned a Severity Assessment Code (SAC), which is a composite indicator of severity and probability of recurrence. Extreme severity incidents are assigned a SAC 1; high severity incidents a SAC 2; medium severity incidents a SAC 3; and low severity incidents a SAC 4. Any employee can report an incident in IIMS, notifiers can remain anonymous if they wish (except if reporting a workplace injury), reporting is entirely voluntary and a report can be lodged if the incident is either witnessed directly by an employee or if he/she hears about the incident second hand.

### Data source and analysis

We reviewed IIMS records describing workplace violence perpetrated against Justice Health staff by patients, correctional officers or patients’ visitors. The three-year study period was from 1 July 2007 to 30 June 2010. Data on workplace violence perpetrated against Justice Health staff by a fellow health worker (also known as horizontal violence) are captured in another information management system and were not included in this study.

We extracted all workplace incidents related to workplace violence (“Incident Type” = “Aggression-Victim” in IIMS). During the study period, Justice Health employed an IIMS administrator. One of the responsibilities of this employee was to maximise IIMS data quality, which involved, among other things, ensuring that incidents were assigned the appropriate “Incident Type”. The following information was obtained from the extracted records: type of violence (physical, verbal or sexual abuse) (in IIMS, incidents that include both verbal and physical abuse are coded as physical abuse); location and time of the incident; age, gender and profession of the victim; status of the aggressor (that is, whether they were a patient, a visitor of a patient or a correctional officer); severity of the incident (SAC); adverse health outcome experienced by the victim (if any) (this may include “mental stress”, which is self-reported by the victim); time taken off work to recover from the incident (if any); and help sought following the incident. In addition, we reviewed the free text description of the incident to identify day-to-day activities that may attract aggression from patients, correctional officers and/or visitors. Each free text description was reviewed and a coding scheme developed (by AWC), which included descriptions of a range of workplace duties. The coding scheme was subsequently applied to each record in order to identify ‘high risk’ work duties.

All of the IIMS records that were extracted described an incident of workplace violence and therefore none were excluded from analysis. Data were analysed using SAS version 9.2 [42]. Descriptive statistics were calculated to describe and summarise records. Significance testing was conducted to determine if experiences of workplace violence varied significantly by victim gender (chi-square test) and victim age (t-test). The study was approved by the Justice Health Human Research Ethics Committee.

## Results

During the study period, 208 incidents of workplace violence were recorded in IIMS: 72 in the 2007–08 financial year; 50 in the 2008–09 financial year; and 86 in the 2009–10 financial year. The victim was verbally abused (with no physical contact) in 71% (n=148) of the incidents, and in 20 of these cases the aggressor threatened to kill the victim. Incidents of physical abuse were less common (27%, n=56). Only 2% (n=4) of the incidents involved a sexual assault (these were subsequently analysed as “physical abuse”).

### Location and time of violent incidents

Almost half (44%, n=92) of the incidents of workplace violence (including both verbal and physical abuse) occurred in an adult male prison (Table 1). One-quarter (n=52) of the incidents occurred in a maximum security adult male prison. Information on the incident location was missing in one record. The type of violence experienced varied by workplace setting, with verbal abuse most commonly encountered by employees who were working in adult male prisons (55%, n=81) and physical abuse most commonly encountered by employees who were working in the forensic hospital (50%, n=30) (Table 1). Relatively few incidents of physical abuse occurred in adult male prisons (18%, n=11).

**Table 1:** **Incidents of workplace violence reported by Justice Health employees between 1 July 2007 and 30 June 2010 by victim’s work setting**^1^

	**Workplace violence**		**Physical abuse**		**Verbal abuse**	
	**n**	**%**	**n**	**%**	**n**	**%**
**Adult male prison**	92	44.4	11	18.3	81	55.1
**Forensic hospital**	40	19.3	30	50.0	10	6.8
**Female prison**	27	13.0	8	13.3	19	12.9
**Prison hospital**	17	8.2	8	13.3	9	6.1
**Juvenile justice centre**	11	5.3	1	1.7	10	6.8
**Police holding cell**	11	5.3	2	3.3	9	6.1
**Other**	9	4.4	0	0.0	9	6.1
**Total**	207	100	60	100	147	100

1. Data source: Justice Health, Incident Information Management System.

Frequency missing (n = 1).

During the three years under review, the average number of incidents of workplace violence recorded in IIMS each month was 5.8. The most incidents occurred in July (16%, n=34) and the least occurred in February (4%, n=9).

The highest proportion (40%, n=84) of incidents occurred between 6:01am and 12 noon, followed by between 12:01pm and 6pm (34%, n=70), between 6:01pm and 12 midnight (23%, n=47), and between 12:01am and 6am (3%, n=6). Information on the time of day of the incident was missing in one record. Most (80%, n=118) incidents of verbal abuse occurred during daylight hours (6am-6pm), compared with 60% (n=36) of incidents of physical abuse. Physical abuse was nearly twice as likely as verbal abuse (33% vs. 18%) to occur in the evening (6pm – 12am).

### Victim and aggressor characteristics

Most (90%, n=187) of the victims of workplace violence were employed as a nurse (Table 2). Two-thirds (66%, n=134) of the victims were females and 34% (n=70) were males. Information on the victim’s sex was missing in four records. About three-quarters (74%, n=107) of the incidents of verbal abuse were perpetrated against a female member of staff. A significantly higher proportion of males than females (55% vs. 25%, X^2^=16.14, p < 0.001) were the victims of physical abuse.

**Table 2:** **Incidents of workplace violence reported by Justice Health employees between 1 July 2007 and 30 June 2010 by victim’s profession**^1^

	**Workplace violence**		**Physical abuse**		**Verbal abuse**	
	**n**	**%**	**n**	**%**	**n**	**%**
**Nurse**	187	89.9	57	95.0	130	87.8
**Medical doctor**	11	5.3	2	3.3	9	6.1
**Allied health**	5	2.4	1	1.7	4	2.7
**Administration**	3	1.4	0	0.0	3	2.0
**Other**	2	1.0	0	0.0	2	1.4
**Total**	208	100	60	100	148	100

1. Data source: Justice Health, Incident Information Management System.

The average age of victims was 43.1 ;years (median 44.7 ;years). One-third of the victims (n=62) were aged 50 ;years and older (Table 3). Information on the victim’s age was missing in 18 records. Victims experiencing physical abuse were significantly younger in age than those experiencing verbal abuse (37.9 ;years vs. 45.1 ;years, T=4.28, p < 0.001).

**Table 3:** **Incidents of workplace violence reported by Justice Health employees between 1 July 2007 and 30 June 2010 by victim’s age**^1^

	**Workplace violence**		**Physical abuse**		**Verbal abuse**	
	**n**	**%**	**n**	**%**	**n**	**%**
**<30 ;years**	32	16.8	17	30.9	15	11.1
**30-39 ;years**	40	21.1	15	27.3	25	18.5
**40-49 ;years**	56	29.5	15	27.3	41	30.4
**50+ years**	62	32.6	8	14.6	54	40.0
**Total**	190	100	55	100	135	100

1. Data source: Justice Health, Incident Information Management System.

Frequency missing (n = 18).

About one-third (32%, n=65) of the incidents of workplace violence occurred while a health professional was either preparing or dispensing medication (Table 4). Forty nine of the records did not clearly specify the work activity the victim was performing at the time of the incident and two records did not include a free text description of the case. Preparing or dispensing medication attracted verbal abuse in particular (40%, n=59). By contrast, the most (33%, n=20) incidents of physical abuse occurred while a health professional was attempting to calm and/or restrain an aggressive patient (Table 4).

**Table 4:** **Activity victim was engaged in when the violence occurred**^1^

	**Workplace violence**		**Physical abuse**		**Verbal abuse**	
	**n**	**%**	**n**	**%**	**n**	**%**
**Preparing or dispensing medication(s)**	65	31.5	6	10.0	59	40.4
**Patient consultation**	33	16.0	9	15.0	24	16.4
**Restraining or trying to calm aggressive patient(s)**	21	10.2	20	33.3	1	0.7
**Observing or supervising a patient(s)**	16	7.8	10	16.7	6	4.1
**Dental treatment**	11	5.3	2	3.3	9	6.2
**Other**	11	5.3	1	1.7	10	6.9
**Unclear/unable to code**	49	23.8	12	20.0	37	25.3
**Total**	206	100	60	100	146	100

1. Data source: Justice Health, Incident Information Management System.

Study period: 1 July 2007 – 30 June 2010. Frequency missing (n = 2).

Most (93%, n=192) of the incidents of workplace violence were initiated by a prisoner/patient. The remainder of the incidents were initiated by either a correctional officer (5%, n=11) or a visitor (2%, n=4). Information on the source of the violence was missing in one record.

### Severity of violent incidents

About half (52%, n=107) of the incidents of workplace violence recorded in IIMS were assigned a SAC 4 (low severity), 46% (n=94) were assigned a SAC 3 (medium severity), 2% (n=5) were assigned a SAC 2 (high severity) and none were assigned a SAC 1 (extreme severity). Information on SAC was missing in two records. Overall, physical abuse was assessed as being more severe than verbal abuse, with 66% (n=38) of incidents of physical abuse being allocated a SAC 3 and a similar proportion (61%, n=90) of incidents of verbal abuse being allocated a SAC 4.

### Adverse health outcomes among the victims of violence

The victim experienced an adverse health outcome (either physical injury, mental stress or both) in 85% (n=154) of the incidents of workplace violence. Information on this variable was missing in 27 records. Mental stress was the most common adverse health outcome and was experienced by 69% (n=106) of victims whose health was negatively affected by the incident. Of the victims who experienced mental stress, most (85%, n=90) had been verbally abused (with no physical contact). Among the 60 victims of physical abuse, 68% (n=41) sustained a physical injury from the incident. Very serious injuries, such as bone fracture (n=1) and concussion (n=1), were rare, although 44% (n=18) experienced bruising and/or laceration(s). There were no workplace deaths during the study period.

Few (11%, n=19) of the victims of workplace violence took time off work in order to recover from the incident. Three per cent (n=5) of the victims were away from work for at least one week. Information on whether the victim took time off work was missing in 39 records.

### Help seeking behaviours of victims

Victims sought help from a health professional or medical assistance (including basic first aid) in 23% (n=39) of the incidents of workplace violence (Table 5). Information on victim help seeking behaviour was missing in 39 records. In line with the assessments of severity described above, 70% (n=37) of the victims of physical abuse either did not require medical attention or required basic first aid (Table 5). Only 6% (n=7) of victims of verbal abuse sought assistance from a health professional (Table 5). Staff counselling was offered to 92% (n=141) of the victims of workplace violence (information on this variable was missing in 54 records).

**Table 5:** Medical treatment sought by victim following an incident of workplace violence

	**Workplace violence**		**Physical abuse**		**Verbal abuse**	
	**n**	**%**	**n**	**%**	**n**	**%**
**Nil**	130	76.9	21	39.6	109	94.0
**First aid**	17	10.1	16	30.2	1	0.9
**Own doctor**	12	7.1	8	15.1	4	3.4
**Emergency department**	8	4.7	8	15.1	0	0.0
**Staff health service**	2	1.2	0	0.0	2	1.7
**Total**	169	100	53	100	116	100

^1^Data source: Justice Health, Incident Information Management System.

Study period: 1 July 2007 – 30 June 2010. Frequency missing (n = 39).

## Discussion

During the three years under review, 208 incidents of workplace violence were recorded in IIMS. Verbal abuse was substantially more common than physical abuse. The most incidents of workplace violence (including both verbal and physical abuse) occurred in an adult male prison, although the most incidents of physical abuse occurred in the forensic hospital. About three-quarters of the incidents of workplace violence occurred during daylight hours (6am-6pm). Most of the victims were nurses and two-thirds were females. Younger employees and males were most likely to be a victim of physical abuse. Preparing or dispensing medication and attempting to calm and/or restrain an aggressive patient were identified as ‘high risk’ work duties. Most of the incidents of workplace violence were initiated by a prisoner/patient. Almost all of the incidents received either a medium or low severity SAC, with more than half assessed to be low severity. Few victims of workplace violence incurred a serious physical injury – there were no workplace deaths during the study period. However, mental stress was common, especially among the victims of verbal abuse. Few victims of verbal abuse sought help from a health professional.

There are four potential limitations of our study. First, some of the variables investigated, such as whether staff counselling was offered to the victims of workplace violence, had a relatively high number of missing values. The proportions calculated for these variables should therefore be interpreted with care. Second, incidents of horizontal violence (Justice Health staff abusing fellow Justice Health staff) were not included in this review. Horizontal violence is a significant problem among some health professions [22,43] (especially nursing [19,20]) and recent research suggests that it is particularly prevalent among correctional health workers [23]. This source of violence should be included in future secondary analyses of workplace violence in correctional health services. Third, some of the IIMS records that were included in this review may have inaccuracies, which introduces the potential of information bias in the findings. For example, an incident may be recorded as physical abuse when, in fact, no physical contact occurred. Fourth, due to factors such as variability in the views of staff about what constitutes a reportable incident [4,44,45] and the perception held by many nurses that violent patients are under significant stress and are therefore not fully responsible for their behaviour [46], it is likely that the 208 incidents recorded in IIMS during the three-year study period are an underestimate of the true incidence of workplace violence among the study population. This is supported by our recent work surveying levels of violence among Justice Health staff, which found that 264 survey respondents had experienced at least one violent incident in their workplace during a three-month recall period, and that patients/prisoners were the primary source of aggression [23] (the quarterly average number of violent incidents recorded in IIMS during the study period was 17.4). Under-reporting of workplace violence is problematic for myriad reasons. For example, accurate, detailed and timely reporting of incidents can inform the establishment of tailored preventive programs [4,44,45]. Table 6 provides an overview of the opinions of some Justice Health staff who participated in the afore-mentioned survey [23] regarding the factors that influence the reporting behaviours of correctional health services employees. An important factor was that some staff considered verbal abuse to be common in the workplace and not serious enough to warrant reporting.

**Table 6:** **The views of Justice Health employees on the factors that influence reporting of incidents of workplace violence**^**1**^

**Theme**	**Participant quote**
***Verbal abuse is part of the job***	
1. Some staff felt that verbal abuse was understandable given the nature of incarceration and therefore preferred to handle this form of abuse informally.	*“As I was aware that the verbal abuse was a reflection of the patient’s frustration with systems and therefore not really directed at me”“I felt no need to report the incident as the outburst was from a patient. The anger was purely associated with their current situation, not because of something I did”*
2. Some did not consider verbal abuse serious enough to report. These staff felt that only threats to their physical safety warranted reporting.	*“I did not consider that the incident warranted recording in IIMS* (Incident Information Management System (IIMS))*. I did not feel my safety was at risk”“You get used to verbal abuse. I would only report physical violence”*
3. Some staff felt that verbal abuse occurred regularly and, as a result, they had become desensitised to it and did not think about reporting it.	*“Verbal abuse is not uncommon in this environment and is quietly forgotten”“I’m desensitised to moderate forms of verbal abuse”*
***Discontent with the management of incidents of workplace violence***	
1. Some staff felt that there was poor follow up of recorded incidents.	*“There was no feedback on the report, no action taken. Does anyone read them? It’s a waste of my time”“The patients and DCS staff* (employees of Corrective Services NSW) *never get spoken to. There are no ramifications. If there are, we never get feedback”*
2. Some staff reported that they were discouraged from reporting an incident involving a correctional officer by their line manager.	*“We are not allowed to* (report an incident in IIMS) *if it involves an officer. We have been told by upper management not to, never”“I brought up my problems with management and I found that they supported non-Justice Health employees more than their own”*
3. Some were concerned that they would be disciplined by their manager if they reported an incident.	*“Although IIMS is supposed to be a system for improving incidents and not a forum for punishment, I find that in most instances it’s the latter. Therefore, if I don’t report it, there are no repercussions for me”“Too much trouble, and can lead to more troubles if you report an officer”*
***Practical barriers to reporting***	
1. Some staff mentioned that they were busy undertaking work duties and therefore did not have the time to complete the *IIMS* form, which they considered time consuming.	*“IIMS takes too long to complete and is cumbersome. Takes too long in a busy workplace”“The workload is too heavy to spend the time to fill in IIMS, and I’m not spending my own time to fill it in”*
2. Some were not clear on the differing functions of IIMS and formal grievance procedures (the latter are used to address horizontal violence).	*“No point reporting on IIMS when it’s the manager yelling. She has access to IIMS”“Passive aggressive behaviour is hard to report on IIMS. Things like being left off team emails, not included in decision making processes, left feeling alone, ostracized does not report well on IIMS”*

1. Data source: Cashmore AW, Indig D, Hampton SE, Hegney DG, Jalaludin BB: Workplace abuse among correctional health professionals in New South Wales, Australia. Australian Health Review 2012, 36:184–190.

The extent of under-reporting of workplace violence described above is consistent with other studies of health professionals [4,21,33,44,45] and highlights the difficulty in using routinely collected administrative data to assess levels of workplace violence. Nonetheless, these data are useful in exploring the characteristics of violent incidents [47]. Our finding that verbal abuse was more common than physical abuse is consistent with a number of previous studies [11,12,23,26,29,48]. This pattern might be explained, in part, by the fact that most health professionals employed by Justice Health practice in a prison health clinic [35], and that, in NSW, prison health clinics are highly secure environments (due to measures such as the requirement that correctional officers supervise patient consultations, the mandatory use of duress alarms by Justice Health staff and the administration of punishments to prisoners who are caught harming a health worker), which limits opportunities for patient-initiated physical abuse [23]. For more on the factors influencing the risk of physical abuse among health professionals who practice in prison health clinics in NSW, see Cashmore et al. [23]. The relatively high incidence of verbal abuse observed may also be explained by the fact that 90% of Justice Health employees are females [35]. It has been suggested that social norms dictate the types of violence aggressors are willing to level at female health professionals, such that it is acceptable to perpetrate verbal abuse against a female but not physical abuse [11]. This assertion is supported by our finding that, despite making up just 10% of Justice Health staff [35], males were more than twice as likely as females to be physically abused.

Care should be taken when comparing levels of workplace violence among the different health care settings within Justice Health. The various environments in which Justice Health staff work vary, sometimes subtly and sometimes significantly, in terms of facility structures, security measures, patient cohorts and other factors that can influence the risk of workplace violence. Bearing this in mind, our finding that the most incidents of physical abuse occurred in the forensic hospital should be interpreted with caution. The forensic hospital differs to most of the health services provided by Justice Health, including primary care delivered in prison health clinics, in terms of the model of care adopted, the physical layout of the facility, the security measures employed and the mental health and treatment needs of the patients who receive care there. These differences pose a unique set of challenges in preventing workplace violence among staff who work in forensic settings. As such, the levels of violence found among employees of the Justice Health forensic hospital are best compared with the findings of studies of workplace violence conducted in forensic settings, particularly studies that have used a secondary analysis design. Daffern and colleagues [33] retrospectively reviewed documented incidents of violence in an 80-bed forensic psychiatric hospital in the Australian state of Victoria and found that, in the hospital’s first year of operation, 56 incidents of violence against staff occurred. This number was more than the 40 incidents that occurred in the 135-bed Justice Health forensic hospital during a period of 17 ;months (the forensic hospital opened in February 2009). The difference observed could be due to a number of factors, such as differences in the reporting cultures of the respective facilities. It should be noted that it is possible that teething problems in establishing the Justice Health forensic hospital’s policies and procedures in relation to staff occupational health and safety contributed to some of the incidents of physical abuse that occurred. 

Our finding that a majority of the victims of workplace violence were employed as a nurse is consistent with previous research [8,16,23,27] and reflects the fact that nurses comprise about two-thirds of all Justice Health staff and an even higher proportion (about 80%) of staff who have direct contact with prisoners/patients [35]. Common nursing activities that were found to be associated with workplace violence include preparing medication or dispensing it, which attracted verbal abuse in particular, and attempting to calm and/or restrain an aggressive patient, which attracted physical abuse in particular. Targeted preventive strategies, such as the on-going training of mental health nurses in the control and restraint of aggressive patients and increasing the correctional officer to prisoner ratio in and around the “pill window” in correctional facilities, may assist in reducing levels of workplace violence among correctional health professionals. However, the impact of such strategies will likely be small unless they form part of a broader, multi-layered program that addresses the varied organisational and environmental determinants of workplace violence in correctional settings [32,49]. Increasing the amount and quality of incident reporting, through activities such as streamlining reporting processes (where needed), ensuring there is synergy between incident management procedures and policies of “Zero Tolerance” and ensuring that the outcomes of and actions arising from investigations of incidents are fed back to the victims [4,23], would aid in identifying and addressing the unique structural and environmental factors that influence the risk of workplace violence among correctional and forensic health professionals.

Our finding that more than half of the incidents of workplace violence received a low severity SAC and that few victims of physical abuse incurred a serious physical injury suggest that delivering health care in a correctional environment may not be as dangerous as one might intuitively think, at least in relation to a practitioner’s physical safety. Although we have argued that under-reporting of workplace violence is likely a problem among Justice Health employees, it is widely held that, in health care settings, the more severe violent incidents are more commonly reported than the less severe incidents [4,44]. It is therefore likely that the assessments of severity and descriptions of physical injuries outlined in this report are reasonably accurate. In order to further reduce the likelihood of severe incidents occurring, correctional health services managers, administrators, health workers and other employees need to maintain vigilance in implementing, evaluating and continuously refining occupational health and safety policies and procedures.

Although we found that serious physical injuries were rare, a high proportion of the victims of workplace violence, especially those who had been verbally abused (with no physical contact), experienced mental stress. Only 6% of the victims of verbal abuse sought help from a health professional following the incident, a finding that is consistent with a recently conducted survey of workplace violence among Australian nurses [11], which found that only 3% of the victims of verbal aggression sought help from a counsellor. The survey, conducted by Farrell et al [11], found that about three-quarters (74%) of the victims of verbal aggression responded instead by talking with a work colleague about their experience(s) (68% found this strategy helpful). It is possible that many correctional health professionals respond to workplace violence, whether it is physical or non-physical abuse, in a similar way. Victims may also debrief with their line manager, a family member or a friend. The actions of correctional health professionals and other employees of correctional health services in response to being abused at work require further study.

Our finding that 92% of the victims of workplace violence were offered staff counselling suggests that the post incident support component of the NSW Ministry of Health and Justice Health policy “Zero Tolerance Response to Violence in the NSW Health Workplace” [39] is being adhered to reasonably effectively, although there remains room for improvement, with the ideal being that *all* staff are offered post incident counselling [39].

## Conclusions

Among employees of a large correctional health service, verbal abuse in the workplace was substantially more common than physical abuse. The most incidents of workplace violence occurred in adult male prisons. Review of the types of adverse health outcomes experienced by the victims of workplace violence and the assessments of severity assigned to violent incidents suggests that, compared with health care settings in the community, correctional settings are fairly safe places in which to practice. Under-reporting of incidents of workplace violence appears to be a problem among employees of correctional health services. Employers of correctional health professionals need to do more to support their staff to thoroughly report violent incidents. Such supportive action would likely deliver the formative information required to ensure that the local context is considered when wide-reaching state policies such as “Zero Tolerance of Workplace Violence” are implemented at the facility level.

## Competing interests

We have none to declare.

## Authors’ contributions

AWC, DI and SEH conceived of the project. All authors assisted in developing the study design. AWC extracted, cleaned and analysed the data. DI and BJ assisted with data analysis. AWC drafted the report. All authors critically reviewed the draft report, and all authors read and approved the final version of the report.

## Pre-publication history

The pre-publication history for this paper can be accessed here:

http://www.biomedcentral.com/1472-6963/12/245/prepub
